# Supplementary material for: Intraspecific Variability in Trionymus aberrans Goux, 1938 (Hemiptera: Coccomorpha: Pseudococcidae) with Description of the Second-Instar Nymph
Source: Insects. 2023 Dec 13;14(12):945. doi: 10.3390/insects14120945 (PMC10744154; doi:10.3390/insects14120945)
Supplement: Supplementary file 1 [file insects-14-00945-s001.zip › insects-2711240-supplementary.pdf]

Table S1. List of examined microscope slides of adult females of *Trionymus aberrans* Goux, 1938.

Coll. number	Valid Taxon Full Name	Gathering Agent Person Name	Identifier Person Name	Locality	Count. code	Date	N
MNHN 806/a/2	Trionymus aberrans Goux, 1938	Goux	Goux	Marseille	FR	1936-08-01	1
MNHN 806/a/3	Trionymus aberrans Goux, 1938	Goux	Goux	Marseille	FR	1936-08-01	1
MNHN Goux coll.1189	Trionymus aberrans Goux, 1938	Goux	Goux	Rhône	FR	1941-09-02	3
DZUS	Trionymus aberrans Goux, 1938	Ogaza, Koteja	Koteja	Szczawnica	PL	1964-06-22	1
DZUS	Trionymus aberrans Goux, 1938	Ogaza	Ogaza	Tompa	HU	1964-09-09	1
DZUS 1439	Trionymus aberrans Goux, 1938	Ogaza	Ogaza	Sutomore	ME	1965-09-23	1
DZUS 2262, 2263	Trionymus aberrans Goux, 1938	Koteja	Koteja	Kraków	PL	1967-09-05	2
DZUS 3214, 3216, 3217, 3218	Trionymus aberrans Goux, 1938	Koteja	Koteja	Mikoszewo	PL	1969-08-04	4
DZUS 3511, 3512, 3513	Trionymus aberrans Goux, 1938	Koteja	Koteja	Kryspinów	PL	1970-07-27	3
DZUS 26	Trionymus aberrans Goux, 1938	Ogaza	Ogaza	Emlékerdő	HU	1964-09-11	1

DZUS 27	Trionymus aberrans Goux, 1938	Ogaza	Ogaza	Tompa	HU	1964-09-09	1
DZUS	Trionymus aberrans Goux, 1938	Ogaza	Ogaza	Sutomore	ME	1965-09-28	1
DZUS 80	Trionymus aberrans Goux, 1938	Simon	Simon	Przerycie	PL	2005-07-25	1
DZUS	Trionymus aberrans Goux, 1938	Simon	Simon	Brantołka	PL	2005-07-28	2
DZUS	Trionymus aberrans Goux, 1938	Simon	Simon	Tworóg Mały	PL	2005-08-16	2
DZUS	Trionymus aberrans Goux, 1938	Simon	Simon	Stanica	PL	2005-08-17	1
DZUS 24	Trionymus aberrans Goux, 1938	Simon	Simon	Bargłówka	PL	2005-08-22	2
DZUS 23	Trionymus aberrans Goux, 1938	Simon	Simon	Przerycie	PL	2005-08-29	1
DZUS 130	Trionymus aberrans Goux, 1938	Simon	Simon	Ruda Kozielska	PL	2006-07-22	2
DZUS	Trionymus aberrans Goux, 1938	Kalandyk-Kołodziejczyk	Kalandyk-Kołodziejczyk	Klucze	PL	2006-08-01	2
DZUS	Trionymus aberrans Goux, 1938	Kalandyk-Kołodziejczyk	Kalandyk-Kołodziejczyk	Rogoźnik	PL	2006-08-12	1
DZUS	Trionymus aberrans Goux, 1938	Kalandyk-Kołodziejczyk	Kalandyk-Kołodziejczyk	Wojkowice	PL	2006-08-12	1
DZUS	Trionymus aberrans Goux, 1938	Kalandyk-Kołodziejczyk	Kalandyk-Kołodziejczyk	Nowa Wieś	PL	2006-08-15	2
DZUS	Trionymus aberrans Goux, 1938	Kalandyk-Kołodziejczyk	Kalandyk-Kołodziejczyk	Góra Siewierska	PL	2006-08-15	3
DZUS	Trionymus aberrans Goux, 1938	Kalandyk-Kołodziejczyk	Kalandyk-Kołodziejczyk	Bukowno	PL	2006-08-24	2
DZUS	Trionymus aberrans Goux, 1938	Kalandyk-Kołodziejczyk	Kalandyk-Kołodziejczyk	Twardowice	PL	2006-09-19	1
DZUS	Trionymus aberrans Goux, 1938	Kalandyk-Kołodziejczyk	Kalandyk-Kołodziejczyk	Toporowice	PL	2007-08-07	2
DZUS	Trionymus aberrans Goux, 1938	Kalandyk-Kołodziejczyk	Kalandyk-Kołodziejczyk	Dąbrowa Górnicza	PL	2008-07-05	1

DZUS	Trionymus aberrans Goux, 1938	Kalandyk-Kołodziejczyk	Kalandyk-Kołodziejczyk	Ruda Śląska	PL	2010-06-30	1
DZUS	Trionymus aberrans Goux, 1938	Kalandyk-Kołodziejczyk	Kalandyk-Kołodziejczyk	Katowice	PL	2010-08-20	1
DZUS	Trionymus aberrans Goux, 1938	Kalandyk-Kołodziejczyk	Kalandyk-Kołodziejczyk	Ruda Śląska	PL	2010-08-21	1
DZUS	Trionymus aberrans Goux, 1938	Kalandyk-Kołodziejczyk	Kalandyk-Kołodziejczyk	Dąbrowa Górnicza	PL	2010-09-07	1
DZUS	Trionymus aberrans Goux, 1938	Kalandyk-Kołodziejczyk	Kalandyk-Kołodziejczyk	Sławków	PL	2010-09-14	1
DZUS	Trionymus aberrans Goux, 1938	Kalandyk-Kołodziejczyk	Kalandyk-Kołodziejczyk	Częstochowa	PL	2010-09-23	1
DZUS	Trionymus aberrans Goux, 1938	Kalandyk-Kołodziejczyk	Kalandyk-Kołodziejczyk	Twardowice	PL	2010-10-01	1
DZUS	Trionymus aberrans Goux, 1938	Kalandyk-Kołodziejczyk	Kalandyk-Kołodziejczyk	Dąbrowa Górnicza	PL	2010-10-02	1
DZUS	Trionymus aberrans Goux, 1938	Kalandyk-Kołodziejczyk	Kalandyk-Kołodziejczyk	Katowice	PL	2010-11-18	1
DZUS	Trionymus aberrans Goux, 1938	Kalandyk-Kołodziejczyk	Kalandyk-Kołodziejczyk	Ruda Śląska	PL	2011-07-13	3
DZUS	Trionymus aberrans Goux, 1938	Kalandyk-Kołodziejczyk	Kalandyk-Kołodziejczyk	Katowice	PL	2011-09-23	1
DZUS	Trionymus aberrans Goux, 1938	Kalandyk-Kołodziejczyk	Kalandyk-Kołodziejczyk	Katowice	PL	2011-09-25	4
DZUS	Trionymus aberrans Goux, 1938	Kalandyk-Kołodziejczyk	Kalandyk-Kołodziejczyk	Ruda Śląska	PL	2011-09-25	30
DZUS 329, 28, 321	Trionymus aberrans Goux, 1938	Kalandyk-Kołodziejczyk	Kalandyk-Kołodziejczyk		AT	2011-10-02	3
DZUS	Trionymus aberrans Goux, 1938	Kalandyk-Kołodziejczyk	Kalandyk-Kołodziejczyk	Dąbrowa Górnicza	PL	2012-08-27	1
DZUS	Trionymus aberrans Goux, 1938	Kalandyk-Kołodziejczyk	Kalandyk-Kołodziejczyk	Katowice	PL	2012-09-25	1

CZPOL COL	Trionymus aberrans Goux, 1938	Kalandyk-Kołodziejczyk	Kalandyk-Kołodziejczyk	Piekary Śląskie	PL	2011-07-26	3
CZPOL COL	Trionymus aberrans Goux, 1938	Kalandyk-Kołodziejczyk	Kalandyk-Kołodziejczyk	Piekary Śląskie	PL	2015-09-20	3
CZPOL COL	Trionymus aberrans Goux, 1938	Kalandyk-Kołodziejczyk	Kalandyk-Kołodziejczyk	Piekary Śląskie	PL	2015-09-25	3
CZPOL COL	Trionymus aberrans Goux, 1938	Kalandyk-Kołodziejczyk	Kalandyk-Kołodziejczyk	Piekary Śląskie	PL	2016-09-27	4
CZPOL COL	Trionymus aberrans Goux, 1938	Kalandyk-Kołodziejczyk	Kalandyk-Kołodziejczyk	Piekary Śląskie	PL	2017-09-02	7
CZPOL COL	Trionymus aberrans Goux, 1938	Kalandyk-Kołodziejczyk	Kalandyk-Kołodziejczyk	Piekary Śląskie	PL	2020-09-12	10
CZPOL COL	Trionymus aberrans Goux, 1938	Kalandyk-Kołodziejczyk	Kalandyk-Kołodziejczyk	Krajno Pierwsze	PL	2018-08-30	4
CZPOL COL	Trionymus aberrans Goux, 1938	Kalandyk-Kołodziejczyk	Kalandyk-Kołodziejczyk	Krajno Pierwsze	PL	2018-08-31	3
CZPOL COL	Trionymus aberrans Goux, 1938	Kalandyk-Kołodziejczyk	Kalandyk-Kołodziejczyk	Krajno Pierwsze	PL	2018-09-01	3
CZPOL COL	Trionymus aberrans Goux, 1938	Kalandyk-Kołodziejczyk	Kalandyk-Kołodziejczyk	Krajno Pierwsze	PL	2019-09-25	5
CZPOL COL	Trionymus aberrans Goux, 1938	Kalandyk-Kołodziejczyk	Kalandyk-Kołodziejczyk	Bolesław	PL	2012-08-16	5
CZPOL COL	Trionymus aberrans Goux, 1938	Kalandyk-Kołodziejczyk	Kalandyk-Kołodziejczyk	Bolesław	PL	2012-09-12	5
CZPOL COL	Trionymus aberrans Goux, 1938	Kalandyk-Kołodziejczyk	Kalandyk-Kołodziejczyk	Ruda Śląska	PL	2011-08-01	6
CZPOL COL	Trionymus aberrans Goux, 1938	Kalandyk-Kołodziejczyk	Kalandyk-Kołodziejczyk	Ostrava	CZ	2018-08-18	9
	Trionymus aberrans Goux, 1938	Kalandyk-Kołodziejczyk	Kalandyk-Kołodziejczyk	Vienna	AT	2015-10	5
	Trionymus aberrans Goux, 1938	Kalandyk-Kołodziejczyk	Kalandyk-Kołodziejczyk	Vienna	AT	2017-06-20	5

	Trionymus aberrans Goux, 1938	Kalandyk-Kołodziejczyk	Kalandyk-Kołodziejczyk	Piekary Śląskie	PL	2018-07-24	6
	Trionymus aberrans Goux, 1938	Kalandyk-Kołodziejczyk	Kalandyk-Kołodziejczyk	Piekary Śląskie	PL	2019-07-22	3
	Trionymus aberrans Goux, 1938	Kalandyk-Kołodziejczyk	Kalandyk-Kołodziejczyk	Piekary Śląskie	PL	2021-08-22	4
	Trionymus aberrans Goux, 1938	Kalandyk-Kołodziejczyk	Kalandyk-Kołodziejczyk	Bolesław	PL	2017-07-20	2
	Trionymus aberrans Goux, 1938	Kalandyk-Kołodziejczyk	Kalandyk-Kołodziejczyk	Bolesław	PL	2018-08-21	2
	Trionymus aberrans Goux, 1938	Kalandyk-Kołodziejczyk	Kalandyk-Kołodziejczyk	Bolesław	PL	2019-07-10	2
	Trionymus aberrans Goux, 1938	Kalandyk-Kołodziejczyk	Kalandyk-Kołodziejczyk	Bolesław	PL	2021-05-09	8
	Trionymus aberrans Goux, 1938	Kalandyk-Kołodziejczyk	Kalandyk-Kołodziejczyk	Ruda Śląska	PL	2017-07-25	10
	Trionymus aberrans Goux, 1938	Kalandyk-Kołodziejczyk	Kalandyk-Kołodziejczyk	Ruda Śląska	PL	2018-09-04	2
	Trionymus aberrans Goux, 1938	Kalandyk-Kołodziejczyk	Kalandyk-Kołodziejczyk	Ruda Śląska	PL	2020-08-05	1
	Trionymus aberrans Goux, 1938	Kalandyk-Kołodziejczyk	Kalandyk-Kołodziejczyk	Ruda Śląska	PL	2020-10-05	10

coll.–collection; count. code–country code; CZPOL COL–Scale insects of Poland collection (In Polish: Czerwce Polski); DZUS-Department of Zoology, University of Silesia (currently Zoology Research Group); MNHN–Muséum national d'Histoire naturelle, Paris; N-number of microscope slides

country codes: AT–Austria, CZ–the Czech Republic, FR–France, HU–Hungary, ME–Montenegro, PL–Poland, RU–Russia

Table S2. Specimens of *Trionymus aberrans* Goux, 1938 analyzed using the scanning electron microscope (SEM).

Valid Taxon Full Name	Gathering Agent Person Name	Identifier Person Name	Locality	Count. code	Date
Trionymus aberrans Goux, 1938	Kalandyk-Kołodziejczyk	Kalandyk-Kołodziejczyk	Bolesław	PL	2012-08-16
Trionymus aberrans Goux, 1938	Kalandyk-Kołodziejczyk	Kalandyk-Kołodziejczyk	Krajno Pierwsze	PL	2018-09-01
Trionymus aberrans Goux, 1938	Kalandyk-Kołodziejczyk	Kalandyk-Kołodziejczyk	Ostrava	CZ	2018-08-18
Trionymus aberrans Goux, 1938	Kalandyk-Kołodziejczyk	Kalandyk-Kołodziejczyk	Piekary Śląskie	PL	2017-09-02
Trionymus aberrans Goux, 1938	Kalandyk-Kołodziejczyk	Kalandyk-Kołodziejczyk	Piekary Śląskie	PL	2020-08-15
Trionymus aberrans Goux, 1938	Kalandyk-Kołodziejczyk	Kalandyk-Kołodziejczyk	Piekary Śląskie	PL	2020-09-12
Trionymus aberrans Goux, 1938	Kalandyk-Kołodziejczyk	Kalandyk-Kołodziejczyk	Ruda Śląska	PL	2012-08-16

code–country codes: CZ–the Czech Republic, PL–Poland
